# Objectionable Terms

**Published:** 1885-06

**Authors:** 


### Editorial.

### OBJECTIONABLE TERMS.

If dentists expect to receive any consideration as scientific, or professional, or educated men, they should exercise greater care in the use of technical terms, than is too often done. What impression must be left upon the mind of one who has been trained to professional exactness, when he hears those who lay claim to professional knowledge call the pulp of a tooth "the nerve." To any one who knows the character of nerve tissue, it conveys the impression of dense ignorance when a dentist speaks of "the bleeding nerve." To say that "the nerve was exposed" is absurd. When one says that he saw "the nerve in the tooth," he simply makes himself ridiculous, for no one ever did that without a microscope. The PULP is composed mainly of connective tissue, but it *contains* blood-vessels and nerve filaments, and it is the pulp that is referred to when dentists speak of "the nerve of a tooth."

Another gross error is the calling the first permanent molar "the sixth year molar," or more commonly "the six year old molar." The expression is extremely discordant, unscientific, and incorrect. There is a grave grammatical error in employing it as it is commonly used, one that is so egregious that no man possessed of even a common school education would commit it, except from the force of habit, or through inexcusable carelessness. Why will not dentists cultivate correct speech, and use language worthy professional men, calling the organs which it is their special province to treat by their proper names, as first, second, and third permanent, or deciduous molars.

To one who has any knowledge of the modern theories concerning septic diseases and conditions, it sounds very strange to hear intelligent men speak of "bugs" in connection with diseases of the mouth. It cannot be possible that any one who reads even the daily or weekly newspaper can entertain the idea that fungi are of an animal nature; yet we not infrequently hear men who claim to possess some share of intelligence and information, actually speaking of bacteria as if they were ravenous beasts, and talking of the "bug theory" of disease. Such expressions indicate an entire lack of comprehension of one of the most important processes of nature—the fermentation of organic substances through the proliferation of microscopical fungi, the growth of vegetable micro-organisms. Brethren, let us banish all these terms as savoring too much of the "tooth-carpentry" period; as reminiscent of the time when dentists laid no claim to being professional men.

